# The Relationship between Subjective Well-being and Social Support among Jordanian University Students

**DOI:** 10.11621/pir.2022.0204

**Published:** 2022-06-30

**Authors:** Ahmad M. Mahasneh

**Affiliations:** a Department of Educational Psychology, Faculty of Educational Sciences, The Hashemite University, Jordan

**Keywords:** Subjective well-being, social support, university student

## Abstract

**Background:**

Although the interest in subjective well-being has flourished during recent decades, there is a general lack of research into this subject throughout the Arab world, and in the Jordanian academic environment in particular.

**Objective:**

The present study aimed to identify any significant gender differences in the level of subjective well-being, and to examine the relationship between subjective well-being and social support among a sample of Hashemite University students.

**Design:**

The study sample comprised 679 male and female undergraduate students from the Hashemite University chosen by purposive method. The College Student Subjective Well-being Questionnaire and Multidimensional Scale of Perceived Social Support were used to collect the data.

**Results:**

The results showed no significant differences in the level of subjective well-being due to the gender variable, but indicated significant differences between genders in satisfaction with academics and school connectedness. The results also showed a positive relationship between subjective well-being and social support.

**Conclusion:**

The current study contributes to enriching the theoretical literature related to gender differences in the level of subjective well-being of Jordanian university students and to examining the relationship between subjective well-being and social support.

## Introduction

As defined by Moore and Diener (2019), subjective well-being is the ability to engage in life with determination, approaching it as a positive, interesting, and exciting experience without the negative and unpleasant effects of distress, fear, and anxiety. Ryan and Deci (2019) noted that life satisfaction is considered a hedonistic concept by many researchers, because, despite requiring a cognitive evaluation of the individual’s life, subjective well-being is also built on moods, feelings, and attitudes.

But while Ryan and Deci (2017) also defined subjective well-being as a marker for healthy physical and psychological functioning, the positive influence of supportive social contexts helps people sustain an independent and self-reliant lifestyle, and the motivation to fulfill one’s basic needs, maintain self-control and respect, and have the dynamism and vitality needed to make the effort required in daily life. The same team of researchers (Ryan & Deci, 2017; Deci & Ryan, 2016) found that energetic activity promoted and increased the feeling of well-being associated with the ability to function proficiently in social contexts, and was an accepted indicator of a higher level of (eudemonic) well-being than that experienced as the result of the autonomous provision of basic needs. Their 2016 article asserted that maintaining a sense of subjective well-being requires exertion of constant effort in the significant activities the individual chooses, and is consequently defined in research terms as a specific outcome.

Diener et al. (2018) described subjective well-being as a state of life-satisfaction wherein pleasant emotions are the norm and unpleasant emotions a rarity, indicating an individual’s self-evaluation based on both the cognitive and emotional perspective. The cognitive reflects the individual’s evaluation of his degree of satisfaction with life, and the emotional reflects his degree of happiness as a result of the balance between the positive and negative.

Other researchers (Nickerson et al., 2011; Salmela-Aro & Tuiminen-Soini, 2010; Sousa et al., 2018) have discussed subjective well-being in the higher education setting in relation to the student’s educational objectives and ambitions, academic engagement, attendance record, educational track or field of study, academic achievement, and drop-out rate.

Therefore, encouraging and supporting students’ subjective well-being, in addition to being an important end in itself, is a significant factor in academic and professional success. Given these findings, it became imperative to recognize and understand the variables stimulating and supporting subjective well-being in university students. To this purpose, Tay et al. (2014) demonstrated that psychological needs are key elements contributing to subjective well-being, while De Freese and Smith (2014) pointed to social support as another important factor, as it provides the social resources necessary to improving students’ quality of life.

The psychological literature has indicated a lack of consistency in findings on gender differences in subjective well-being (Batz & Tay, 2018). The results of some previous studies (Stevenson & Wolfers, 2009) indicated that males have a higher level of subjective well-being compared to females, while other studies (Fujita et al., 1991) indicated that females have a higher level of subjective well-being. Other studies (Inchley et al., 2020; Shmotkin, 1990; Sagi et al., 2021) showed no differences in the level of subjective well-being due to gender.

Sarason and Sarason (2009) illustrated the impact of social support on social and psychological adjustment, using a range of demonstrative, material, and physical sources gleaned from a person’s contacts with people in their environment. The importance of social support in coping with stress was noted by Cohen (2004) and De Freese and Smith, (2013), whose research specifically showed that individuals who enjoyed strong social support were more capable of coping successfully with the adverse effects of stress. Such support plays a psychologically beneficial role, since the individual does not feel alone and isolated in having to deal with his/her problems. Social support diminishes the weight of the stress-threat felt by the individual, allowing him/her to study and adopt alternative coping-strategies, and thereby boost self-esteem.

A study by Infurna and Jayawickreme (2019) supported the benefit of the interpersonal resources as a constructive overall framework enabling individuals to handle stress in more positive ways. Studies by Diener et al. (2020) and Tov (2018) asserted that social support was the main incentive for an individual’s success, while Sarason and Sarason (2009) defined social support as incorporating personal, familial, and social contacts and interactions. Helsen et al. (2000) pointed out the change that occurs during adolescence, when peer-relationships become a person’s main source of support, and the perception of parental/familial support may remain constant or diminish.

Four types of social support have been identified by researchers: informative, instrumental, emotional, and compatible (Holt-Lunstad, 2018; House, 1981). In recent decades, one of the most vital aspects of an individual’s ability to cope with and surmount stressful problems has been identified as social support: Solid support empowers a healthy lifestyle (Varga & Zaff, 2018) and in the adolescent phase, is seen to reduce aggressive behavior. Ronen et al. (2016), and Ronen and Rosenbaum (2010) identified social support as a key resource in allaying fears of war in adolescents.

It is important to be aware of and understand the factors affecting and associated with the subjective well-being of university students, since there is a general lack of research into this subject throughout the Arab world, and in the Jordanian academic environment in particular. Researchers are thus motivated to conduct studies into subjective well-being in students and expand the corpus of knowledge available in the theoretical literature. The present study aimed to identify whether there are any significant gender-variable differences in the level of students’ subjective well-being, and examine the relationship between subjective well-being and social support among a sample of Hashemite University students.

## Method

### Study Sample

The study sample comprised 679 students at the Hashemite University enrolled in three prerequisite courses during the academic year 2020–2021; they were selected by the available sample method. The participants consisted of 399 male (58.8%) and 280 female students (41.2%); 320 (47%) were from scientific colleges, and 359 (53%) from humanities colleges. There were 159 first year students (23%), 178 second year (26%), 180 third year (27%), and 162 fourth year students (24%); the ages of the study sample ranged between 18–22 years.

### Study scales

#### College Student Subjective Well-being Questionnaire (CSSWQ)

The CSSWQ was developed by Renshaw and Bolognino (2016). It consists of 15 items distributed into four dimensions: 1) satisfaction with academics (3 items); 2) school connectedness (4 items); 3) college gratitude (4 items); and 4) academic efficacy (4 items). The students used a 7-point Likert scale to answer the CSSWQ items:1 = strongly disagree to 7 = strongly agree.

For the purpose of this study, the researcher translated the CSSWQ from English into Arabic; then the CSSWQ items in Arabic and in English were presented to two faculty members in the English Department to ensure the accuracy and integrity of the translation.

To check the validity of the CSSWQ in the current study, the author used the Pearson correlation between the total CSSWQ and its subscale as shown in *Table 1.*

**Table 1:** Pearson correlation between CSSWQ and subscales

Variables	Satisfaction with academics	School connectedness	College gratitude	Academic efficacy	Total scale
Satisfaction with academics	1				
School connectedness	0.86	1			
College gratitude	0.89	0.89	1		
Academic efficacy	0.78	0.80	0.74	1	
Total scale	0.94	0.95	0.95	0.87	1

To check the reliability of the CSSWQ in the current study, the author calculated its internal consistency by using Cronbach’s alpha. The results were 0.77 for the CSS-WQ, and 0.71, 0.72, 0.83 and 0.81 respectively for satisfaction with academics, school connectedness, college gratitude, and academic efficacy.

#### Multidimensional Scale of Perceived Social Support (MSPSS)

The MSPSS was developed by Zimet et al. (1988). It consists of 12 items grouped into three dimensions: 1) family (4 items); 2) friends (4 items); and 3) significant other (4 items). Cronbach’s alpha was 0.88 for the MSPSS. The students used a 7-point Likert scale to answer the MSPSS items: 1 = very strongly disagree to 7 = very strongly agree.

For the purpose of this study, the researcher translated the MSPSS from the English language into Arabic. Then the MSPSS items in Arabic and in English were presented to two faculty members in the English Department to ensure the accuracy and integrity of the translation.

To check the validity of the MSPSS in the current study, the author calculated the Pearson correlation between the total MSPSS score and its subscale as shown in *Table 2.*

**Table 2:** Pearson correlation between MSPSS and subscales

Variables	Family	Friends	Significant other	Total scale
Family	1			
Friends	0.18	1		
Significant other	0.57	0.29	1	
Total scale	0.78	0.59	0.83	1

To check the reliability of the MSPSS results in the current study, the author calculated its internal consistency using Cronbach’s alpha: it measured 0.85 for the MSPSS, and 0.84, 0.79, and 0.81 respectively for family, friends, and significant other.

### Data collection and analysis

The study scales were translated and the accuracy of the translation from the English language and into Arabic was verified. The validity and reliability of the study scales were then confirmed by a pilot sample of 50 students. The study scales were prepared and distributed to the participants using Microsoft Forms. This study was conducted during COVID-19. The students were informed about the study on the official Facebook page of the Hashemite University. They were told that participating in the study was voluntary, and that the data would be used for scientific research purposes only.

The process of data collection took three weeks. The data was checked before analysis to ensure that there were no missing data. Means, standard deviations, and an independent sample t-test analysis were calculated to examine the differences in the level of subjective well-being according to the gender variable, and the Pearson correlation coefficient was calculated to examine the relationship between subjective well-being and social support.

## Results

*Table 3* shows the results from calculation of the mean and standard deviation of the subjective well-being and social support-based gender variables.

**Table 3:** Mean(M) and standard deviation(SD) for subjective well-being according to gender

Variable	Male		Female	
	M	SD	M	SD
Satisfaction with academics	4.80	1.25	5.40	1.19
School connectedness	5.28	1.08	5.06	0.57
College gratitude	3.96	1.57	3.89	0.78
Academic efficacy	5.87	0.84	5.85	0.57
Total scale	4.99	1.16	5.03	0.58

To determine any significant differences in the level of subjective well-being based on the gender variable, an independent sample test analysis was done, with the results shown in *Table 4.*

**Table 4:** Results of Independent Sample t-Test

Variables	Gender	Mean	T	df	Sig
Satisfaction with academics	Male	4.80	–6.290	677	0.00
	Female	5.40			
School connectedness	Male	5.28	3.116	677	0.00
	Female	5.06			
College gratitude	Male	3.96	0.656	677	0.51
	Female	3.89			
Academic efficacy	Male	5.87	0.361	677	0.71
	Female	5.85			
Total scale	Male	4.99	–.502	677	0.61
	Female	5.03			

The results of the independent sample test analysis showed no statistically significant differences in the level of subjective well-being and subscales (college gratitude and academic efficacy) attributable to student gender. But there were statistically significant differences in the level of satisfaction with academics and school connectedness attributable to gender. The mean score of satisfaction with academics for female students was higher than for male students, while the mean score of school connectedness for male students was higher than that for female students.

To examine the relationship between subjective well-being and social support, the Pearson correlations were used, with the results shown in *Table 5.*

**Table 5:** Pearson correlations between subjective well-being and social support

Variables	Family	Friends	Significant other	Social support
Satisfaction with academics	0.49*	0.03	0.38*	0.39*
School connectedness	0.49*	0.02	0.26*	0.35*
College gratitude	0.42*	0.05	0.33*	0.37*
Academic efficacy	0.49*	0.11*	0.27*	0.31*
Subjective well-being	0.50*	0.01	0.33*	0.38*

Note. *p= 0.01.

The results of the Pearson correlations showed a positive correlation between subjective well-being and social support, and a positive correlation between the subjective well-being subscales (satisfaction with academics, school connectedness, college gratitude, and academic efficacy) and social support subscales (family and significant other). But while there was no correlation between the subjective well-being subscales (satisfaction with academics, school connectedness, and college gratitude) and friends, a positive correlation was found between academic efficacy and friends.

## Discussion

The results showed no statistically significant differences in the level of subjective well-being attributable to student gender, an outcome supported by a majority of research studies into juvenile/teenage coping capability either by type or method of response (Coleman & Hagell, 2007). Gender differences were found in the methods used in the students’ assessments and their emotional reactions to traumatic events.

The results showed that the level of satisfaction with academics for females was higher than that for males. This can be explained in light of the high level of academic achievement of females; this author notes through his academic experiences that females always excelled in academic achievement and got the highest scores on achievement tests. In addition, good performance in academic courses and student satisfaction with their academic achievement leads to positive academic experiences for female students at the university, which positively reflects on their feelings of a high level of satisfaction with academics.

The results also showed that the level of school connectedness for males was higher than that for females. This can be explained by the fact that males seek to build social relationships with other students at the university, and participate in various university activities, which fact is then positively reflected on their level of school connectedness.

Research by Gelhaar et al. (2007), Reschly et al. (2008), and Tamres et al. (2002) had similar results: their studies showed a tendency for females to react to events more intensely as well as for their responses to be more emotionally centered and reliant on social support, whereas males were less likely to react to emotional stress.

The results of this study therefore showed a positive relationship between subjective well-being and social support, and indicated that an increase in the level of social support led to an increase in the level of subjective well-being of university students, *i.e.*, the improved level of student ability to make cognitive judgments about his/her life as a whole, and seeing it as positive and satisfactory, while maintaining self-confidence and the ability to meet expectations and achieve his goals. The results can be explained in light of the fact that students with higher social support are more understanding of their strengths and weaknesses, are more flexible and able to accept them, and are happier and more satisfied with their lives, which leads to a higher level of subjective well-being. In addition, social support has a strong effect on the self-system, since it increases self-esteem and self-confidence, as well as a sense of control over difficult situations facing the individual. Thus, social support generates a degree of positive feelings that enables the individual to deal with disturbing external events without feeling the strong negative impact that disables the ability to overcome and control.

Barrera (2000) suggested that social support performs many functions, including guidance and counselling, providing advice and protection from falling into error, and behavioral assistance regarding social functioning. Social support and assistance in the many different situations to which the recipient is exposed, helps to build self-confidence, and develop an individual’s positive attitude toward life. Social support also plays a preventive role, by healing psychological and mental discord and conflict, and also contributes to the individual’s positive attitude and personal development, giving him or her protection against being strongly affected by stress or crises.

Some researchers (Sarason et al., 2001; Zhang et al., 2022) note the key role played by parents in their child’s early years, into adolescence, and as young adults, in the child’s degree of dependence on parental support and advice. The literature illustrates the significance of parental support in building the concept of independence in their children, since it is known to have positive links to the child’s ability to adjust academically, to be tenacious, and have the determination to succeed (Gagnon et al., 2019; Guay, 2022; Ratelle et al., 2021), as well as improving student subjective well-being (Boonk et al., 2018; Chirkov, 2017; Monacis et al., 2021; Niemiec & Ryan, 2009). It is of interest that the effects of parents support for the child’s autonomy with regard to accepting academic choices made by the child, continue into adulthood.

Despite the importance of parental advice and guidance, Collins and Madsen (2006) noted that in an individual’s late teens and early twenties, support from friends, especially in emotional situations, is frequently seen as being more significant than familial advice. García-Moya et al. (2015) noted that, after family relationships, young adults considered friends to be the most important source of support. Help from friends was a major contribution during the transition from school to university (Dixon-Rayle & Chung, 2007; Shaver et al., 1985), and Argyle (2013) in contributing to subjective well-being. Unlike the relationship between parents and child, peer friendship assumes equality, empathy, and affinity. In a study by Surjadi et al. (2011), it was found that the transitional period from adolescence to adulthood saw a gradual replacement of parental influence by that of friends or later, romantic partners. In studies focused on autonomy support, researchers Deci et al. (2006) and Kasser & Ryan (1999) found that psychological well-being was increased by the influential impact of support from friends.

Ronen et al. (2016) designed a study to investigate the relationship between subjective well-being and social support among adolescents. The results of the study showed a positive relationship between subjective well-being and such social support. Ratelle et al. (2013) carried out a study to investigate the relationship between subjective well-being and social support among university students, whose results also showed a positive relationship between subjective well-being and social support. Alcantara et al. (2017) developed a study to investigate the relationship between subjective well-being and social support among children and adolescents, with the same results, as did previous studies by Caserta et al. (2017), Francis et al. (2018), Holliman et al. (2021), Village & Francis (2021), Zeidner et al. (2016), and Zhou & Lin (2016).

## Conclusion

The current study contributes to enriching the theoretical literature related to gender differences in the level of subjective well-being of Jordanian university students, as well to the relationship between students’ sense of subjective well-being and social support. The results showed no significant differences in the level of subjective well-being due to the gender variable, but indicated a positive relationship between subjective well-being and social support. These results recommend conducting future studies that investigate the relationship between subjective well-being and academic self-efficacy.

## Limitations

The present study was limited to a sample of Bachelor degree students at the Hashemite University, as well as the use of the self-report method in the data collection process.
